# Annual Abundance and Population Structure of Two Dung Beetle Species in a Human-Modified Landscape

**DOI:** 10.3390/insects10010002

**Published:** 2018-12-28

**Authors:** Julliana W. Barretto, Carlos A. Cultid-Medina, Federico Escobar

**Affiliations:** 1 Red de Ecoetología, Instituto de Ecología, A.C., Carretera antigua a Coatepec 351, El Haya, Xalapa C.P., Veracruz 91070, Mexico; barrettojulliana@gmail.com; 2 Red de Diversidad Biológica del Occidente Mexicano, Instituto de Ecología, A.C.—Centro Regional del Bajío, Av. Lázaro Cárdenas No. 253, Col. Centro, Pátzcuaro C.P., Michoacán 61600, Mexico

**Keywords:** cloud forest, Cormack-Jolly-Seber, mark-recapture, Scarabaeinae, seasonality

## Abstract

Population studies are essential for understanding different aspects of species’ biology, estimating extinction probability, and determining evolutionary and life history. Using the mark-recapture method, we studied the abundance and population structure of dung beetle species (*Deltochilum mexicanum* and *Dichotomius satanas*) over one year in a human-modified landscape in Mexico. We captured 1960 individuals with a net recapture rate of 11%. *Deltochilum mexicanum* had a higher rate of recapture (14%) than *Dichotomius satanas* (5%). Annual variation in abundance was similar for both species, with maximum abundance occurring in summer and a marked reduction during winter. *Deltochilum mexicanum* was dominant inside the forest, and its abundance was influenced by vegetation cover, temperature, and humidity. *Dichotomius satanas* was more frequent outside the forest, and none of the considered environmental variables affected its abundance. The adult sex ratio of *Deltochilum mexicanum* was female-biased, whereas that of *Dichotomius satanas* was male-biased. The maximum estimated population size was similar for both species, but *Deltochilum mexicanum* had a higher number of new individuals and survival rate. Since species with different biological attributes presented a similar pattern of abundance and population structure, we conclude that environmental conditions are the main regulator of dung beetle populations in the human-modified landscape.

## 1. Introduction

Population structure and dynamics result from the interaction between species’ life-history traits and the environmental characteristics of their habitats. In general, any demographic parameters relevant for population size (age structure, sex ratio, longevity, and survival rate, among others) can be used to evaluate population dynamics and structure [1,2,3]. Both density-independent factors, such as environmental conditions, and density-dependent factors, such as the rate of natality, mortality, and migration, are determinants of population size. Likewise, population size is regulated by the magnitude of intraspecific interactions (e.g., search for mates, parental care, and territorial defense) and interspecific interactions (e.g., competition for resources, parasitism, and depredation) [1,3]. Therefore, understanding the causes of temporal and spatial variation in the number of individuals in a population is important for elucidating the mechanisms that structure populations. In this way, it is possible to adequately evaluate the vulnerability of species to the modification of environmental conditions at both a local and landscape scale [4,5,6].

An extensive literature has examined the seasonality of poikilothermic organisms like insects and has also documented the large diversity and complexity of morphological, physiological, and behavioral adaptations that enable such organisms to endure seasonal adversity (e.g., low temperatures and drought) [7,8,9]. Several variables, especially temperature and humidity, are considered determinants of the life cycle, development rate, and demographic structure of insects [10,11,12]. Generally, in tropical regions with little seasonal variation, population size and structure are expected to be similar throughout the year [13,14]. Species often reproduce year-round and present a multivoltine reproductive behavior with generational overlap and relatively stable population size over time [15]. However, according to Wolda (1988) [7], this does not necessarily mean that temporal variations are not present in populations of tropical regions [8]. On the other hand, in markedly seasonal environments (temperate, arid, or semi-arid regions), the population structure of species is expected to change throughout the year and to be synchronized with changes in climatic conditions and resource availability [15]. This results in fluctuations in the entry and exit of individuals in a population and, consequently, variations in population size [13].

In addition to temporal changes, insect population size and structure vary in space [16]. For example, at the landscape scale, differences in vegetation structure, microclimate, and resource supply may create different environmental conditions for individuals of the same population [17]. The vast majority of insects show variation in life-history traits at the population level depending on environmental conditions. Changes may be observed in the period of diapause, pupal size, the start of the breeding season, or in the distribution of adults among different habitat types [18,19]. In turn, population studies have shown that landscape characteristics can regulate rates of fertility and survival, which also depend on population density and the quality of available habitats. As a result, differential population growth rates and persistence may be evident at the landscape scale [10,16].

Much of the information available for dung beetles, an insect group recognized as ecological indicators of human disturbance [20], has come from studies at the community level, and information at the population level is limited to a few species [21,22]. Currently, most published population studies about dung beetles have been carried out in relatively small areas over short periods of time (<8 months) covering a single climatic season, mainly the rainy season [23,24,25], limiting our comprehension of the population ecology of this group of insects. It is useful to understand the ecological processes that operate at a population level for evaluating species-specific responses to environmental changes and for identifying the life-history traits associated with extinction risk [26,27,28].

The objective of the present study was to evaluate changes in the abundance and population structure of two dung beetles with differing natural history traits, *Deltochilum mexicanum* (Burmeister, 1848) and *Dichotomius satanas* (Harold, 1867), over 12 months in a human-modified landscape. Both species are typical of Mexican cloud forest, which is considered a conservation priority because it shelters a high diversity and a high number of endemic species [29]. Both beetle species are large in size (>10 mm), which facilitates their marking, and are relatively abundant throughout the year [30,31]. Specifically, *D. mexicanum* is a copronecrophagous and nocturnal roller species, generally associated with well-preserved forested areas [32,33]. Meanwhile, *D. satanas* is a coprophagus and nocturnal tunneler species mostly found in open and semi-open areas altered by human activities (e.g., secondary forests, orchard crops, and pastures) [30,31,34]. Using mark-recapture data, we aimed to answer the following questions: (1) How does the abundance of *D. mexicanum* and *D. satanas* vary throughout the year in two types of vegetation cover (forested and non-forested) in a human-modified landscape? (2) How does the population structure of *D. mexicanum* and *D. satanas* vary throughout the year in regard to their age and sex ratios? And, finally, (3) how do the estimates of population size, survival rate, and the number of new individuals for *D. mexicanum* and *D. satanas* change over one year at the landscape scale? 

## 2. Material and Methods

### 2.1. Study Area

The study was carried out on the eastern slope of Cofre de Perote to the west of the city of Xalapa, Veracruz, Mexico. The climate is temperate and humid year-round. The average annual precipitation varies between 1500 and 2000 mm and the average temperature between 19 and 17 °C [35]. Three seasons can be distinguished: A cold and dry season from November to March ($\overset{´}{X}\;$± SD: 16 ± 1.7 °C, precipitation: 283 mm), a warm and dry season from April to May (21 ± 0.6 °C, 173 mm), and a rainy season from June to October (19.4 ± 0.7 °C; 1131 mm) [36]. The vegetation type of this region corresponds with cloud forest [37].

**Figure 1:**
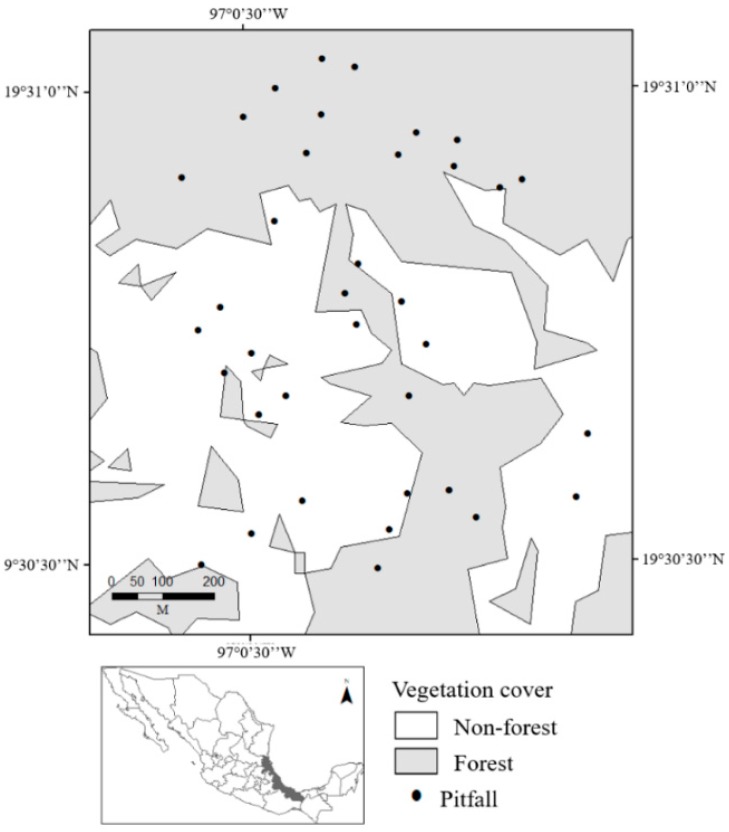
Spatial distribution of the pitfall traps used to sample populations of *Deltochilum mexicanum* and *Dichotomius satanas* over one year (August 2015 to July 2016) in a human-modified cloud forest landscape in Veracruz, Mexico.

The sampling was performed in a landscape of ~126 ha (1.26 × 1.16 km; 19°30′55.81″ N, 97°0′19.88″ W) with an elevation range of 1520 to 1780 masl (Figure 1). The landscape is composed of well-preserved cloud forest (50%), secondary vegetation (23.5%), pastures (24%, with and without trees), and commercial tree plantations (2.5%). In the present study, the vegetation covers were categorized into two types: Forested areas (F) or non-forested areas (NF, including land uses associated with human activities). This landscape exemplifies the current state of the Mexican cloud forest, which mostly exists within a mosaic of different land uses as a result of agricultural expansion and urban growth. Only 1% of the original distribution of cloud forest remains [36].

### 2.2. Mark-Recapture of Dung Beetles

The mark-recapture of dung beetles was carried out between August 2015 and July 2016. In total, 36 non-lethal pitfall traps were used: 16 traps were placed in forested areas and 20 in non-forested areas. All traps were separated by a distance of at least 50 m (Figure 1). Each trap consisted of a 1L disposable plastic container buried at the ground level with a plastic funnel secured in the upper portion to prevent beetles from escaping [38]. Traps baited with 45 g of human excrement were placed for 24 h capture periods, leaving a day between sampling events to guarantee the free dispersion of the marked individuals. This resulted in five effective sampling days (120 h in total) per month. The individuals captured in each event were marked with a consecutive number that was scratched on the surface of the pronotum or elytron with a Mototool (Dremel Stylus 1100^®^, Dremel, Mexico) following the protocol of Martínez-Quintero et al. [39]. At the end of each 24 h period, marked individuals were released next to the trap of capture. The sex of each individual was determined, and the dates of mark-recapture, trap location, and cover type (F or NF) where each individual was captured and recaptured were recorded. The relative age of each individual (two categories: Teneral or recently emerged and non-teneral adult individuals) was also determined based on the degree of sclerotization of the exoskeleton, which is soft, flexible, and pale in color in recently emerged individuals [40].

### 2.3. Environmental Variables

Temperature and humidity were measured in the study area with six data loggers (iButton^®^, Maxim Integrate, San Jose, CA, USA). Three were installed in forested sites and three in non-forested sites, covering most of the landscape. The data loggers were programmed to record the variables every 10 min and remained in the field throughout the entire sampling period except during the month of August 2015. Additionally, accumulated monthly precipitation was obtained from the closest meteorological station located ~3.4 km from the study area. These data are available on the National Online Portal for Transparency (Portal Nacional de Transparencia) of the National Institute for Transparency, Access to Information, and Personal Data Protection (Instituto Nacional de Transparencia, Acceso a la Información y Protección a Datos Personales).

### 2.4. Data Analysis

#### 2.4.1. Annual Variation in Abundance and Environmental Variables

To examine variation in the abundance of the two species throughout the year, population curves were constructed [1] for the landscape and for each cover type (F and NF). At the landscape level, observed abundance was expressed in two ways: (1) As the total number of individuals captured per month, or total abundance (N_total_), and (2) as the net number of individuals, or net abundance (N_net_ = N_total_ − number of individuals recaptured per month). For cover type, the curves were constructed considering only net abundance (N_net_). The behavior of these curves in addition to available knowledge on the natural history of each species provides information on the structure and temporal variation of populations, including voltinism type, breeding cycle duration, and oviposition periods [1].

To establish whether the temporal variation in the abundance of each species was related to cover type (F and NF), generalized linear models (GLMs) were fit to the data. N_net_ was modeled with respect to month and cover type (fixed factors). Besides the null model, four models were specified per species, three with respect to (i) month; (ii) cover type; (iii) month + cover type, and (iv) a complete model including the interaction between month and cover type. For the model fit, a negative binomial distribution was used (g(x) = 1/x), which allows overdispersion to be reduced in cases where count response variables are associated with a high frequency of zeros [41,42]. The selection of the most suitable model was based on the significance of the model, the AICc values (second-order Akaike information criterion for small samples <40) [43], the normality of the residuals (Shapiro test, α = 0.05), and the overdispersion coefficient (ĉ < 1.5) [41]. In addition, the relationship between N_net_ and monthly accumulated precipitation, temperature, and humidity was evaluated. For each variable, a simple linear regression was fit to the data, and the fit of each model was then evaluated based on the normality of the residuals. All models were fit using R code [44].

#### 2.4.2. Population Structure

The age ratio (AR) was calculated as the number of non-teneral individuals/number of teneral individuals. The adult sex ratio (ASR) was calculated considering only non-teneral adults as follows: Male/male + female. The resulting value of the ASR is 0.5 if the number of males and females is equal [45]. This method is the most recommended for estimating sex ratios in nature given that it considers individuals as discrete units and thereby reflects the relative abundance of each sex in a population [46]. As the expected ASR is 0.5 (1:1), we used chi-square (χ^2^) tests to determine if the observed ASR statistically differed from the expected ratio.

Using the Cormack-Jolly-Seber (CJS) method [47,48], three population parameters were estimated per mark-recapture event for each species: Population size ($\hat{N}$*i*), survival rate ($\hat{\Phi }$*i*), and the number of new individuals or recruitment rate (birth + immigration) ($\hat{B}$*i*). Since it is not possible to predict or establish the trends of population parameters beyond the evaluated year using the CJS method, the results and discussion are focused on the maximum and minimum values of the estimations, highlighting the possible causes of variation in these values. The CJS method is appropriate for estimating parameters in open populations and does not overestimate the amplitude of the confidence intervals [49]. The population parameters were estimated by FSA package 0.4.3 using R code [44,50].

## 3. Results

During the sampling year, 66 mark-recapture events were carried out in which 1960 individuals were marked (1260 *D. mexicanum* and 700 *D. satanas*). Considering both species, the net recapture rate (i.e., single recapture) was 11% (214 individuals). *Deltochilum mexicanum* presented almost three times more single recaptures (14%, 176 individuals) than *D. satanas* (5%, 38 individuals). With respect to multiple recaptures, 22 individuals of *D. mexicanum* were recaptured two times, and five were recaptured three times. Only two individuals of *D. satanas* were recaptured two times. For both species, the minimum time interval between capture and recapture was 48 h, and the maximum interval was 365 days for *D. mexicanum* and 306 days for *D. satanas*.

### 3.1. Annual Variation in Abundance and Environmental Variables

**Figure 2:**
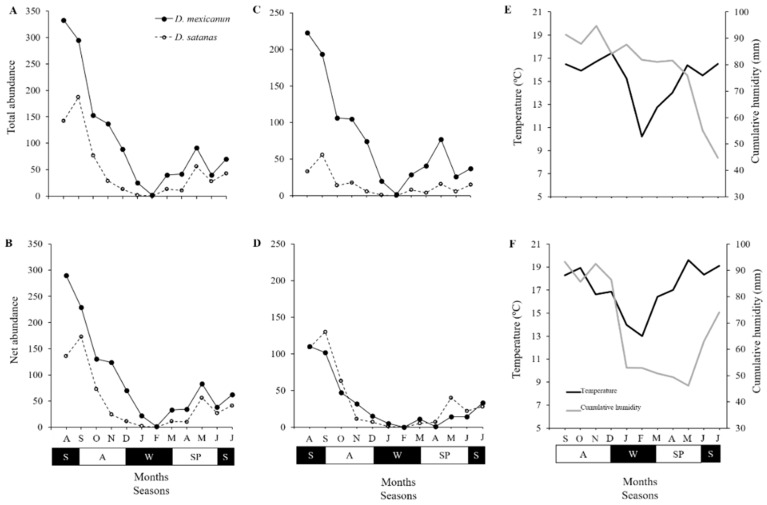
Population curves of *Deltochilum mexicanum* and *Dichotomius satanas* in a human-modified cloud forest landscape in Veracruz, Mexico. (**A**) Population curves of total abundance (N_total_: Total number of individuals captured per month) and (**B**) net abundance (N_net_: Total number of individuals captured per month − number of individuals recaptured per month). In addition, species populations curves considering the type of vegetation cover, forested (**C**) or non-forested (**D**); and the annual variation in temperature (°C) and cumulative humidity (mm) in forested areas (**E**) and non-forested areas (**F**) are shown. Seasons: S = summer, A = autumn, W = winter, and SP = spring.

The seasonal variation in the abundance pattern of abundance was similar for both species. The abundance curves presented a peak between the end of summer (August) and the beginning of autumn (September) followed by a reduction in captures until the end of winter (February) when no individuals were captured. In the following months, a slight increase in the number of individuals of both species was observed and maintained with some fluctuations until the beginning of summer (June 2016) (Figure 2A,B).

*Deltochilum mexicanum* was more abundant in forested areas (888 individuals, 70%) than in non-forested areas (372 individuals, 30%), whereas *D. satanas* presented the opposite pattern (forest: 181 individuals, 26%; non-forest: 519 individuals, 74%). In forested areas, the *D. mexicanum* population presented two peaks in abundance: One during the transition from summer to autumn (between August and September) and another of smaller magnitude near the end of spring (May) (Figure 2C). The abundance of *D. satanas* in forested areas was relatively low (≤50 individuals/month) yet constant throughout the year (Figure 2C). In non-forested areas, both species followed a similar pattern: The number of individuals rapidly decreased toward the end of the year but increased once again toward the end of winter (March) (Figure 2D).

At the landscape level, the N_net_ of both species differed with respect to month and type of vegetation cover, and the interaction between these latter two factors was not significant (Figure S1, Table S1). The N_net_ of *D. mexicanum* was linearly and positively related with precipitation at the landscape scale (R^2^ = 0.19; *P* = 0.026), with temperature in the forested areas (R^2^ = 0.30; *P* = 0.052), and with humidity in the non-forested areas (R^2^ = 0.50; *P* = 0.009) (Table S1). The net abundance of *D. satanas* was not related to any of the variables in either type of vegetation covers (Table S1).

### 3.2. Population Structure

**Figure 3:**
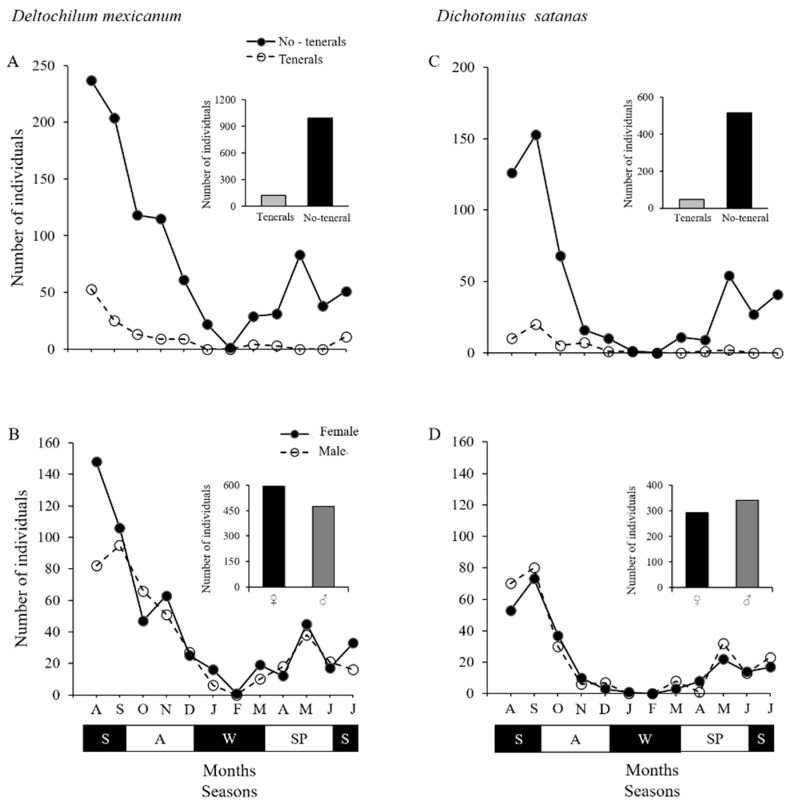
Annual variation in the number of teneral and non-teneral individuals (**A**,**C**) and in the number of males and females in *Deltochilum mexicanum* and *Dichotomius satanas* populations (**B**,**D**) in a human-modified cloud forest landscape in Veracruz, Mexico. Seasons: S = summer, A = autumn, W = winter, and SP = spring.

For the populations of both species, the frequency of teneral individuals was low, representing 13% and 8% of the *D. mexicanum* (AR = 6.67) and *D. satanas* (AR = 11.10), respectively. This pattern was maintained throughout the year (Figure 3A,C). The largest number of teneral individuals of both species was found during the transition between summer and autumn (between August and September) (Figure 3A,C).

The ASR of *D. mexicanum* was significantly female-biased (♂ 475 and ♀ 594, ASR = 0.44; χ^2^ = 13.2; *P* < 0.05, Figure 3B). However, in the months of October, April, and June, the number of males surpassed the number of females (Figure 3B). The ASR of *D. satanas* was male-biased (♂ 342 and ♀ 293, ASR = 0.53; χ^2^ = 3.7; *P* = 0.05; Figure 3D). Males were more numerous than females toward the end of the year and in the months of March, May, and July (Figure 3D).

**Figure 4:**
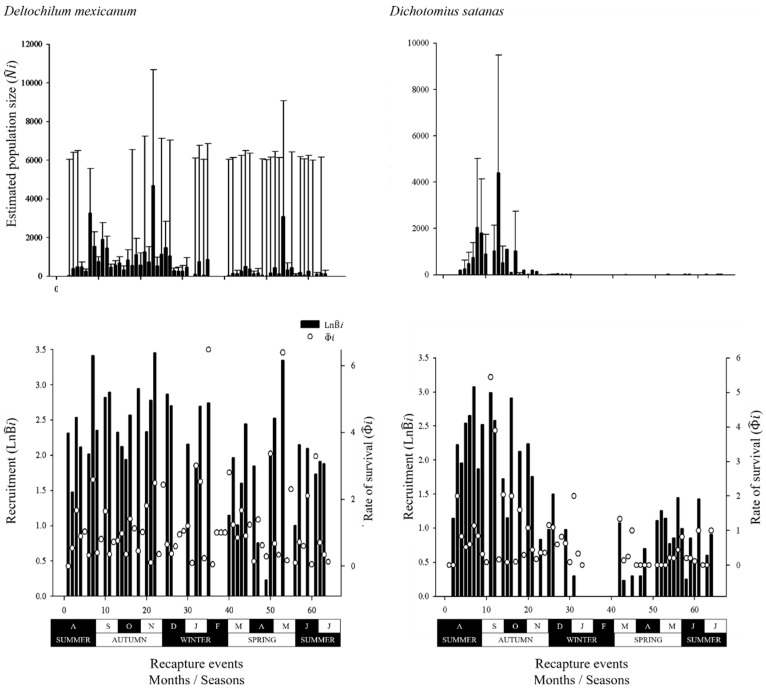
Estimated population size ($\hat{N}$*i* ± 95% CI), recruitment ($\hat{B}$ *i*, (birth + immigration) ± 95% CI), and rate of survival ($\hat{\Phi }$ *i*) estimated for *Deltochilum mexicanum* and *Dichotomius satanas* populations based on 66 mark-recapture events over one year (August 2015–July 2016) in a human-modified cloud forest landscape in Veracruz, Mexico. Recruitment ($\hat{B}$ *i*) was transformed by ln (Ln$\;\hat{B}$*i*) to facilitate comparison

The maximum estimated population size ($\hat{N}$*i*) was similar between species (*D. mexicanum* = 4687 individuals and *D. satanas* = 4395 individuals). The estimated number of new individuals and the rate of survival were higher for *D. mexicanum* ($\hat{B}$*i* = 2.6; $\hat{\Phi }$*i* = 6.48) than for *D. satanas* ($\hat{B}$*i* = 1.2; $\hat{\Phi }$*i* = 5.44). For both species, the estimates for the three parameters varied considerably over the course of the 66 mark-recapture events; the highest values were recorded in autumn and the lowest values in winter (Figure 4).

## 4. Discussion

Our study represents an initial effort to understand seasonal variation in the population structure of two dung beetles, *D. mexicanum* and *D. satanas*, in a human-modified landscape in the cloud forest region of Mexico, one of the most threatened ecosystems in the country. For both species, high recapture rates (5% and 14%) were achieved [21], and it was possible to describe changes in abundance and breeding patterns and to estimate the demographic parameters, such as population size, survival rate, and recruitment rate. Our results are of great value considering that, recently, dung beetles were included on the Red List of Threatened Species by the International Union for Conservation of Nature (http://www.iucnredlist.org). Currently, for 80 percent of the species in this list, information on the status and tendency of populations is unknown.

The annual abundance patterns of *D. mexicanum* and *D. satanas* were characterized by a maximum between the end of summer and beginning of autumn followed by a strong reduction in the number of individuals during winter. This suggests that both species have a univoltine reproductive behavior and a short breeding season that is restricted to the warm and rainy season [1]. According to Hanski and Cambefort [51], the adult feeding behavior required to reach sexual maturity and the search for mates also occur during seasons with optimal environmental conditions. Several studies suggest that the life cycle of dung beetles, especially the nesting and emergence of new individuals (tenerals) is primarily modulated by temperature and precipitation as well as the availability of food [40,52,53]. Our results and those observed in the tropical dry forest [54,55] suggest that the activity of dung beetles decreases considerably during the most climatically severe seasons of the year, such as during the dry season or when temperatures are low. During these periods, beetles probably enter into states of physiological inactivity below ground [56,57] and, therefore, the detection of individuals decreases considerably. For this reason, studies on the population dynamics and structure that document temporal variation in abundance over long periods (>1 year) are necessary to understand the effects of climate variation and the modification of habitats in both the medium and long term.

The results also suggest that the type of vegetation cover is a key factor that explains the distribution of species at the landscape level. We confirmed, similar to several previous studies at the community level, that *D. mexicanum* prefers well-conserved cloud forest areas [32,33], whereas *D. satanas* prefers modified environments, including secondary vegetation, coffee plantations, and pastures [30,31,34]. The preference of dung beetle species for a determined type of environment is essentially a response to microclimatic variables, which are driven by structural vegetation characteristics [58] and the composition and configuration of the landscape [32,59,60]. However, several Scarabaeinae species have a high dispersion capacity (~2 km in 48 h) and can use extensive portions of the landscape [25,61]. For this reason, population studies that consider more extensive landscapes are necessary to identify how environmental attributes affect the spatial-temporal dynamics and persistence of populations.

Although much of our knowledge on the reproductive biology of Scarabaeinae comes from laboratory studies, our results coincide with the known reproductive biology of several species belonging to the genera *Deltochilum* and *Dichotomius* [40,56]. The maximum recorded number of individuals and the presence of a single peak of the emergence of tenerals per year suggest that *D. mexicanum* and *D. satanas* have a larval development phase of approximately 12 months given that the period between oviposition and emergence of new individuals corresponds with that of larval development. This has also been reported for other species of dung beetles with similar life-history traits (i.e., univoltine, large in size, and inhabit seasonal environments) [57,62,63,64]. From these results, we can also infer that both species have lifetimes equal to or greater than 12 months. Previous records for 24 dung beetle species under laboratory conditions indicate that adult longevity varies from 1.5 to 24 months [65,66]. Such reproductive biological characteristics correspond with those of *K*-strategist species, a trait considered typical of dung beetles, which prioritize reproductive efficiency to guarantee the survival of offspring [40].

An ASR biased toward one of the sexes suggests that environmental conditions differentially affect males and females [67,68]. Differential mortality is common in species like dung beetles in which the sexes have distinct nutritional requirements and energy expenditures as a result of differential mobility and investment in parental care [68]. In these cases, the ASR may be biased toward the sex with lesser probability of death [69,70,71]. Although we do not know with certainty which factors determined the sexual proportions of the studied species, an ASR biased towards the females, as observed for *D. mexicanum*, is a characteristic of populations that present a high level of spatial aggregation where males do not compete for the females [72]. Meanwhile, an ASR biased toward males, as observed for *D. satanas*, has been linked to increased intrasexual competition for resources and couples, which could lead to a highly segregated population in space [73].

With respect to the maximum estimated population size, both species presented a similar population density at the landscape level (*D. mexicanum*: 37 individuals/ha; *D. satanas*: 35 individuals/ha). This suggests that *D. mexicanum* and *D. satanas* have similar reproductive potential. The maximum estimated population size for both populations is high (≥1000 individuals) and guarantees the persistence of both populations in the landscape in the long term [2]. Our results agree with other population studies on dung beetles carried out in modified Neotropical landscapes in which estimated population sizes varied between 5000 and 25,000 individuals [25,74,75].

The comparison of the maximum estimates for new individuals and the survival rate enables us to assume that the processes of entry and exit of individuals distinctly contribute to the population dynamics of each species. For example, *D. satanas* has a greater capacity of movement in the landscape, including movement through modified areas [76], thus the migration of individuals appears to strongly contribute toward the population dynamics of this species. Meanwhile, *D. mexicanum* is restricted to forested areas with lower capacity to movement [76], thus the probability of finding mates during the reproductive season could be greater, which results in a high entry of new individuals in the population via birth. However, to confirm these hypotheses, more detailed demographic studies are necessary at larger spatial and temporal scales.

In conservation biology, populations are the unit of primary interest, and understanding the structure and dynamics of insect populations helps to establish the bases for practical conservation measures at the species level [77]. Our results contribute to establishing the conservation status of two dung beetles based on two biological parameters of interest for conservation: Changes in abundance over time and population size [77]. The *D. mexicanum* population could be more vulnerable to habitat loss than *D. satanas* because of its close association with forest areas and lower mobility in the landscape. Such information could be useful for functional connectivity models in order to identify critical elements of the landscape for the management and conservation of cloud forest species. Nonetheless, population studies at the landscape scale are necessary to identify the long-term responses of dung beetles to habitat modification and climate change.

Information is lacking on many aspects of the population ecology of dung beetles in tropical regions. Although the abundance of both dung beetle species in this study clearly fluctuated with seasonal changes, to understand the magnitude of these changes based on the recommendations of Hanski [78], future studies on the population ecology of dung beetles should (i) implement monthly sampling for at least three years; (ii) perform long-term sampling over extensive geographical areas (>100 ha); (iii) take into account innovations in mark-recapture methods for medium- and small-sized species (<10 mm); (iv) aim to explain the specific responses of different species at the population level to environmental changes resulting from the modification of ecosystems and climate, and (v) simultaneously evaluate temporal variation in the population structure and the reproductive phenology of dung beetles.

## 5. Conclusions

Our results demonstrated that the population structure and dynamics of the studied dung beetle are modulated at the local scale by environmental conditions, and are associated with seasonal variation. Differences in the life-history traits of species, including habitat preferences, reproductive behavior, food relocation behavior, nesting behavior, and the capacity for movement, appear to play an important role in the population structure and in demographic processes (i.e., mortality, migration, and birth) that are determinant of population dynamics.

## Supplementary Materials

The following are available online at http://www.mdpi.com/2075-4450/10/1/2/s1. Figure S1: Temporal variation in the mean number of individuals (±95% CI) of *Deltochilum mexicanum* and *Dichotomius satanas* populations considering the type of vegetation cover (forest or no-forest) in a human-modified cloud forest landscape in Veracruz, Mexico. Seasons: S = summer, A = autumn, W = winter, and SP = spring; Table S1: Generalized linear models (GLM) with a negative binomial distribution and simple linear models (Lm) for evaluating the relationship between the net abundance (N_Net_) of *Deltochilum mexicanum* and *Dichotomius satanas* and environmental variables at a landscape level, and type of vegetation cover (forested or non-forested) in a human-modified cloud forest landscape in Veracruz, Mexico. Significant or selected models in bold. N_Net_ = net abundance of species. Cover = cover type (forested or non-forested). Mon = months of sampling. AP = accumulated monthly precipitation (mm). T = monthly temperature (T °C). H = monthly relative humidity (% RH).
