# Supplementary material for: Annual Abundance and Population Structure of Two Dung Beetle Species in a Human-Modified Landscape
Source: Insects. 2018 Dec 28;10(1):2. doi: 10.3390/insects10010002 (PMC6358878; doi:10.3390/insects10010002)
Supplement: Supplementary file 1 [file insects-10-00002-s001.pdf]

insects
MDPI
Article

Annual Abundance and Population Structure of Two Dung Beetle Species in a Human-Modified Landscape

Julliana W. Barretto [1], Carlos A. Cultid-Medina [2,*] and Federico Escobar [1,*]

1 Red de Ecoetología, Instituto de Ecología, A.C., Carretera antigua a Coatepec 351, El Haya, Xalapa C.P. 91070, Veracruz, Mexico; barrettojulliana@gmail.com
2 Red de Diversidad Biológica del Occidente Mexicano, Instituto de Ecología, A.C.—Centro Regional del Bajío, Av. Lázaro Cárdenas No. 253, Col. Centro, Pátzcuaro C.P. 61600, Michoacán, Mexico
* Correspondence: carlos.cultid@inecol.mx; federico.escobarf@gmail.com

Received: 6 November 2018; Accepted: 27 December 2018; Published: date

Supplementary Material

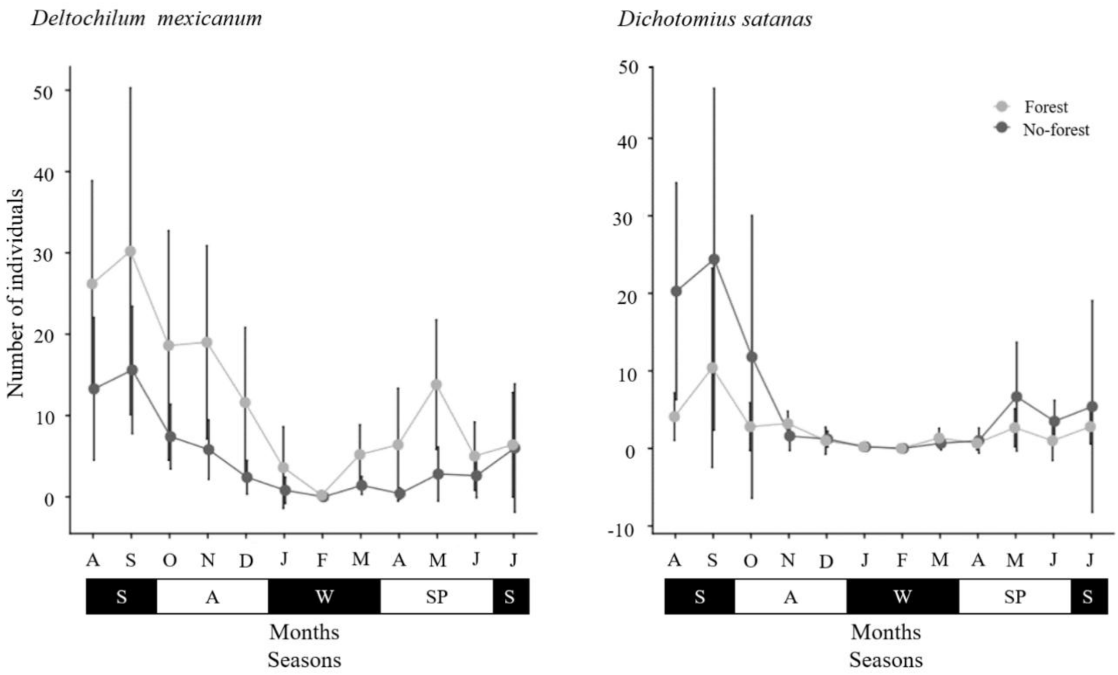

Figure S1. Temporal variation in the mean number of individuals (±95% CI) of *Deltochilum mexicanum* and *Dichotomius satanas* populations considering the type of vegetation cover (forest or no-forest) in a human-modified cloud forest landscape in Veracruz, Mexico. Seasons: S = summer, A = autumn, W = winter, and SP = spring.

Table S1. Generalized linear models (GLM) with a negative binomial distribution and simple linear models (Lm) for evaluating the relationship between the net abundance (N_{Net}) of *Deltochilum mexicanum* and *Dichotomius satanas* and environmental variables at a landscape level and type of vegetation cover (forested or non-forested) in a human-modified cloud forest landscape in Veracruz, Mexico. N_{Net} = Net abundance of species. Cover = cover type (forested or non-forested). Mon = months of sampling. AP = accumulated monthly precipitation (mm). T = monthly temperature (T °C). H = monthly relative humidity (% RH).

Species	Analysis level	Variables	Model (Interaction between variables)	P–Shapiro	AICc	ĉ	R^2	P	F
D. mexicanum	Landscape	Vegetation cover	Null model	0.0000	2048.88				
			GLM (N_{net} ~ cover)	0.0053	850.45	1.1737			
			GLM (N_{net} ~ cover*mon)	0.0048	780.87	1.3041			
			**GLM (N_{net} ~ cover+mon)**	**0.25**	**764.93**	**1.2071**			
		**Accumulated precipitation**	**Lm (N_{net} ~ AP)**	**0.0716**	**223.71**		**0.19**	**0.0259**	**5.788**
	Forested area	**Temperature**	**Lm (N_{net} ~T)**	**0.24**	**118.07**		**0.27**	**0.0517**	**5.022**
		Humidity	Lm (N_{net} ~H)	0.6621	120.49		0.11	0.1678	2.251
	Non-forested area	Temperature	Lm (N_{net} ~T)	0.0200	105.07		0.17	0.1173	3.001
		**Humidity**	**Lm (N_{net} ~H)**	**0.3075**	**99.362**		**0.50**	**0.0086**	**11.17**
D. satanas	Landscape	Vegetation cover	Null model	0.00	1865.7				
			GLM (N_{net} ~ cover)	0.00	667.16	1.0717			
			GLM (N_{net} ~ cover*mon)	0.35	638.68	1.2315			
			**GLM (N_{net} ~ cover+mon)**	**0.07**	**619.8**	**1.1302**			
		Accumulated precipitation	Lm (N_{net} ~AP)	0.0014	208.89		0.22	0.0167	6.810
	Forested area	Temperature	Lm (N_{net} ~T)	0.0011	96.09		0.09	0.1904	2.005
		Humidity	Lm (N_{net} ~H)	0.0075	97.850		−0.06	0.5554	0.375
	Non-forested area	Temperature	Lm (N_{net} ~T)	0.0002	114.68		0.23	0.0786	3.930
		Humidity	Lm (N_{net} ~H)	0.23	115.51		0.17	0.1178	2.999

P–Shapiro—the normality of the residuals using Shapiro test; AICc—second-order Akaike information criterion for small samples < 30; Ĉ—overdispersion coefficient.
